# APOBEC3-Related Editing and Non-Editing Determinants of HIV-1 and HTLV-1 Restriction

**DOI:** 10.3390/ijms26041561

**Published:** 2025-02-12

**Authors:** Sharee Leong, Hesham Nasser, Terumasa Ikeda

**Affiliations:** 1 Division of Molecular Virology and Genetics, Joint Research Center for Human Retrovirus Infection, Kumamoto University, Kumamoto 860-0811, Japan; 2 Graduate School of Medical Sciences, Kumamoto University, Kumamoto 860-8556, Japan

**Keywords:** APOBEC3 family proteins, retrovirus restriction, HIV-1, HTLV-1, deaminase-dependent mechanisms, deaminase-independent mechanisms

## Abstract

The apolipoprotein B mRNA editing enzyme catalytic polypeptide-like 3 (APOBEC3/A3) family of cytosine deaminases serves as a key innate immune barrier against invading retroviruses and endogenous retroelements. The A3 family’s restriction activity against these parasites primarily arises from their ability to catalyze cytosine-to-uracil conversions, resulting in genome editing and the accumulation of lethal mutations in viral genomes. Additionally, non-editing mechanisms, including deaminase-independent pathways, such as blocking viral reverse transcription, have been proposed as antiviral strategies employed by A3 family proteins. Although viral factors can influence infection progression, the determinants that govern A3-mediated restriction are critical in shaping retroviral infection outcomes. This review examines the interactions between retroviruses, specifically human immunodeficiency virus type 1 and human T-cell leukemia virus type 1, and A3 proteins to better understand how editing and non-editing activities contribute to the trajectory of these retroviral infections.

## 1. Introduction

Restriction factors (RFs) are intracellular host proteins that inhibit viral replication, providing intrinsic immunity against viral infections (reviewed in [1,2,3,4,5]). RFs are either constitutively expressed or induced by mediators of the innate immune response to counteract specific steps of the viral life cycle (reviewed in [1,3,4,5,6]). The first discovered RF was Friend virus susceptibility-1 (Fv1), which restricts murine leukemia virus infection [7,8]. Afterward, multiple RFs were identified as inhibitors of human immunodeficiency virus type 1 (HIV-1) infection, which they achieve by blocking various stages of the virus’ life cycle (reviewed in [1,2,3,4,5,6]). However, viruses have evolved strategies to evade RF activity, enhancing viral infection, fitness, and spread (reviewed in [1,2,3,4]). Although viruses can escape RF-mediated restriction, rendering these factors ineffective in controlling replication in natural hosts, RFs often exhibit strong antiviral activity against viruses from other species, creating species-specific barriers to infection (reviewed in [1,5,9,10]). This dynamic interplay involves RFs targeting viral proteins to inhibit their functions and viral proteins counteracting RFs to promote replication (reviewed in [1,4,11]), ultimately shaping viral pathogenesis and infection outcomes.

**Figure 1:**
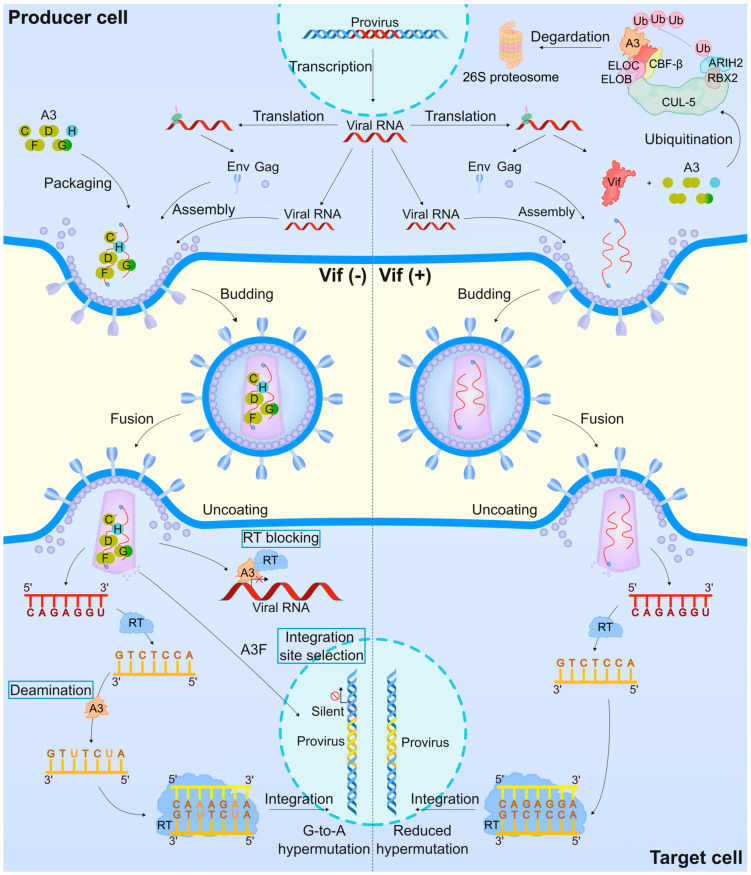
Mechanisms underlying A3-mediated restriction of HIV-1 infection. The A3-mediated restriction of Vif-deficient HIV-1 infection involves the binding of A3 proteins to viral RNA (red), which is then packaged into nascent virions during viral assembly in the producer cells. In target cells, A3 proteins perform deamination-dependent editing on the viral single-stranded cDNA intermediates (brown), introducing potentially lethal G-to-A mutations (highlighted in orange). Additionally, A3 proteins exert a deaminase-independent, non-editing restriction by physically hindering viral reverse transcription and altering the selection of proviral DNA integration sites, favoring transcriptionally silent regions of host DNA. To counteract A3-meditated restriction, HIV-1 employs its accessory protein Vif, which ubiquitinates A3 proteins, targeting them for proteasomal degradation. Consequently, A3 proteins are poorly incorporated into nascent virions, and the cDNA intermediates experience few or no sublethal G-to-A mutations, resulting in a reduced restriction of HIV-1 replication. Each A3 Z domains are colored, respectively, light green: Z2 domains, dark green: Z1 domain, and blue: Z3 domain. A3: apolipoprotein B mRNA editing enzyme catalytic polypeptide-like 3/APOBEC3, ARIH2: Ariadne homolog 2, CBF-β: Core-binding factor β, Cul-5: Cullin 5, ELOB: Elongin B, ELOC: Elongin C, Env: Envelope, Gag: Group-specific antigen, RBX2: RING-box protein 2, RT: Reverse transcriptase, Ub: Ubiquitin, Vif: Viral infectivity factor.

**Figure 2:**
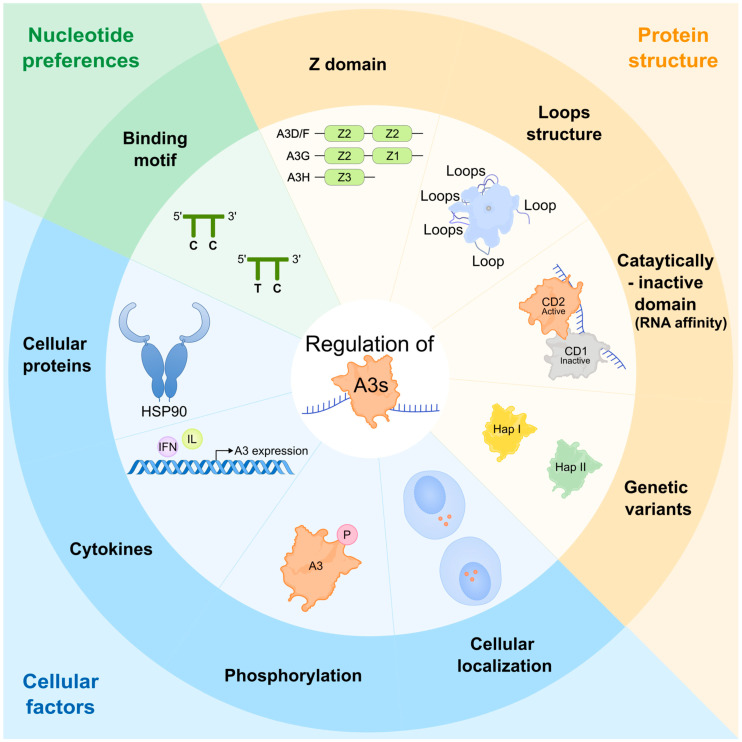
Structural, cellular, and substrate-based regulation of A3 family protein activity. The activity of A3 family proteins is influenced by various factors, including their protein properties, domain organization, amino acid composition, and conserved secondary structural features near the catalytic site, which affect substrate selection and regulate deamination enzymatic activity. Genetic variants of individual A3 proteins can lead to variable deamination activity. The presence of preferred nucleotides in the substrates enhances the deamination activity of specific A3 proteins. Cellular factors, such as protein localization, phosphorylation status, and the influence of cellular cofactors (e.g., HSP90) or upstream regulators (e.g., IFNs), further modulate the activity of A3 proteins, influencing both their editing and non-editing functions.

The human apolipoprotein B mRNA editing enzyme catalytic polypeptide-like 3 (APOBEC3/A3) family of proteins is a critical group of RFs that defend against viral infections and suppress endogenous retroelement activity (reviewed in [1,2,12,13,14,15,16,17]). In humans, the A3 family comprises seven members: A3A, A3B, A3C, A3D, A3F, A3G, and A3H (Figure 1) (reviewed in [2,18,19,20]). The genes of this family are arranged as a tandem cluster between the flanking genes *CBX6* and *CBX7* on chromosome 22 (reviewed in [20,21,22]). A3 enzymes catalyze cytosine (C) deamination to uracil (U) in single-stranded DNA (ssDNA) substrates (reviewed in [2,18,19,21]). The deamination mechanism relies on a conserved zinc-binding domain (Z domain) and occurs via zinc-mediated hydrolysis, where a zinc-stabilized hydroxide ion interacts with the four-position of C, replacing the amine group (NH_2_) with a carbonyl group (reviewed in [22,23]). A3 proteins possess either one (A3A, A3C, and A3H) or two (A3B, A3D, A3F, and A3G) conserved Z domains, categorized into three distinct groups: Z1, Z2, and Z3 (Figure 2) (reviewed in [2,19,20,24,25]). These structural differences underpin the functional diversity of A3 proteins.

Most functional insights into A3 proteins as RFs come from studies on retroviruses, particularly HIV-1 (reviewed in [1,2,18,22]). A3G was the first A3 protein identified to restrict the infectivity of viral infectivity factor (Vif)-deficient HIV-1 [26], binding viral and cellular RNA, packaging into nascent virions from virus-producing cells (Figure 1), and inducing lethal C-to-U mutations in viral cDNA (reviewed in [1,2,18,22]). Other A3 proteins, including A3C-I188, A3D, A3F, and stable haplotypes of A3H, also restrict HIV-1 infection in CD4^+^ T lymphocytes [27,28,29,30,31,32,33]. Additionally, A3 proteins employ deaminase-independent mechanisms to exert antiviral activity (Figure 1) [34,35,36,37,38,39,40,41]. To counteract A3 protein activity, HIV-1 has evolved a Vif-mediated proteasomal degradation pathway that prevents A3 proteins from being packaged into viral particles (reviewed in [1,2,18,22,42]).

**Figure 3:**
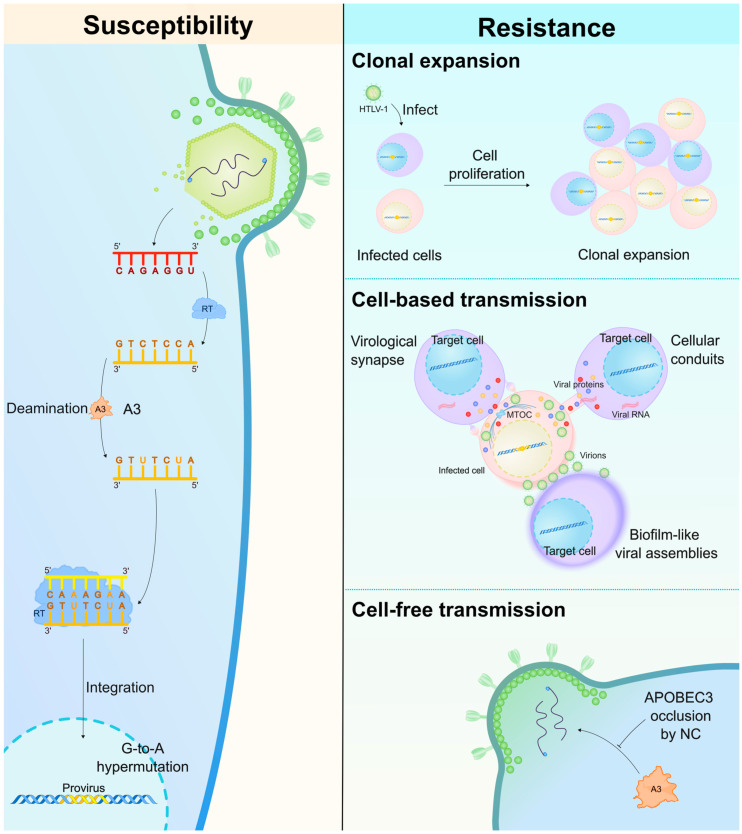
Controversial outcome of the A3–HTLV-1 interaction. During HTLV-1 reverse transcription, A3 proteins can induce editing activity (**left**) via A3-mediated deamination. A3G, for instance, induces G-to-A mutations in the proviruses of HTLV-1 carriers and patients with ATL. Overexpression studies also suggest that A3A, A3B, and A3H stable haplotypes can restrict HTLV-1 infection. However, HTLV-1 appears to be relatively resistant to A3 protein activity due to its distinct replicative strategy and transmission methods (**right**). The integrated HTLV-1 genome drives the clonal expansion of infected CD4^+^ T cells (**top right**), promoting viral dissemination without the production of large numbers of viral particles. HTLV-1 uses cell-based transmission mechanisms (**middle right**), including viral synapses, tunneling nanotubes, and cellular protrusions, minimizing exposure to A3 proteins. Finally, the HTLV-1 NC protein (**bottom right**) impairs A3G packaging into HTLV-1 virions, further contributing to the virus’ resistance to A3-mediated restriction.

Human T-cell leukemia virus type 1 (HTLV-1), another retrovirus, is also targeted by A3 proteins. Although HIV-1 and HTLV-1 share similar replication cycles, HTLV-1 spreads primarily through the clonal expansion of infected cells rather than via the production and release of new virions, thereby reducing exposure to A3-mediated antiviral effects (Figure 3) (reviewed in [43,44,45]). Consequently, the selective pressure exerted by A3 proteins on HTLV-1 differs markedly from that on HIV-1. This review explores the mechanisms through which A3 proteins antagonize HIV-1 and HTLV-1, along with the countermeasures these viruses employ to evade A3 protein activity, ultimately shaping infection outcomes.

In addition to retroviruses, many reports have demonstrated an interaction between A3 family proteins and non-retroviruses as A3-induced mutational signatures were detected in the genomes of ssDNA virus (transfusion-transmitted virus [46]), double-stranded DNA viruses (human herpes viruses [47,48], human papillomavirus [49,50], human polyomaviruses [50,51], human orthopoxvirus [52,53,54,55]), hepadnavirus (hepatitis B virus [50,56,57,58,59]), and single-stranded RNA (ssRNA) viruses (human coronaviruses [50,60,61,62,63,64]). Furthermore, the antiviral activity of A3 family proteins is also extended to parvovirus [65,66], herpes simplex virus 1 [47], Epstein-Barr virus [48], hepatitis B virus [67,68], and human coronavirus NL63 [69]. Although A3 family proteins have been reported to contribute to the evolution of severe acute respiratory syndrome coronavirus 2 (SARS-CoV-2) [50,60,61,62,63,64], these proteins may support SARS-CoV-2 replication [63,70].

## 2. Physiological Functions of APOBEC Family Proteins

The A3 proteins form part of the broader APOBEC family, which has been identified in most vertebrates [71,72,73,74,75]. The first member discovered was APOBEC1 (A1) [76,77], which is abundantly expressed in the small intestine, where it plays a role in lipid metabolism by targeting the apolipoprotein B (*apoB*) mRNA substrate (reviewed in [78,79,80]). Subsequently, several A1 proteins from various nonhuman species were found to deaminate ssDNA [81,82,83] and exhibit restriction potential against retroviruses and endogenous retroelements in cell line models [82,83,84,85,86,87,88,89]. In mice and rabbits, *A1* mRNA is also expressed in immune cells along with the small intestine and liver [82,90]. These findings suggest an innate immune function for A1 protein in combating retroviruses and mobile elements in some nonhuman species [75]. The second identified APOBEC family member was activation-induced cytosine deaminase (AID), which mainly catalyzes the deamination of immunoglobulin (*Ig*) genes [91]. AID protein initiates somatic hypermutation and recombination events that drive antibody class switching (reviewed in [80,92,93]). Although this protein can also bind RNA, it exhibits no catalytic activity in this context [94]. Other APOBEC family members include APOBEC2 (A2) and APOBEC4 (A4) proteins. A2 protein is expressed in cardiac and skeletal muscles and is crucial for muscle development, as demonstrated by the development of myopathy in A2-knockout animal models [95,96]. A4, a less-characterized protein, is primarily expressed in mammalian testes and may contribute to promoter modulation or antiviral responses in birds [97,98]. Importantly, with the exception of A3D, A2, and A4 proteins, other APOBEC family proteins can edit epigenetic codes (reviewed in [99,100]) by deaminating 5-methylcytosine (5mC) to thymine (T). This process produces T-to-guanine (G) mismatches, followed by error-free DNA repair [101,102].

## 3. Factors Regulating the Catalytic Activity of A3 Family Proteins

The catalytic activity of A3 proteins is largely influenced by their biochemical and structural characteristics, as well as interactions with various cellular factors that determine the net outcome of A3-mediated deamination and retrovirus restriction capabilities (Figure 2) (reviewed in [15,19,22,103,104,105]). Understanding these factors is essential to evaluating the efficiency of A3-mediated catalytic activity. This section highlights the regulatory factors influencing A3 family proteins’ catalytic activity, which are key determinants of their restriction effectiveness against retrovirus infections (Figure 2).

### 3.1. Structural Characteristics of A3 Family Proteins

The protein organization of A3 family proteins markedly affects their activity (Figure 2). The catalytic domains of different A3 proteins have evolved distinctly around their conserved Z domains (Figure 2) (reviewed in [2,9,15,20]). Variations in the length, composition, and spatial arrangement of conserved secondary structural features near the catalytic site influence substrate selection and regulation of deamination, resulting in variable activity (Figure 1) (reviewed in [15,103,106,107]).

Interactions between A3 family proteins and nucleic acids occur through a shallow substrate-binding groove defined by four loops around the active site (loops 1, 3, and 7, with minor contributions from loop 5). High-resolution structural analyses of A3 proteins have revealed substantial plasticity and sequence variability within these loops (reviewed in [2,107]). Differences in the amino acid composition, length, and spatial confirmation of loops 1, 3, and 7 contribute to functional variations in substrate recognition and deamination activity (Figure 1) (reviewed in [15,107]). Studies using chimeric proteins, where putative DNA-binding loop regions of A3G protein were replaced with those from A3A protein, showed that replacing loop 3 enhanced A3G catalytic activity without altering its preferred dinucleotide substrate (5′-CC, where the underlined C is the target C). Conversely, replacing loop 7 altered A3G’s dinucleotide preference, mimicking A3A’s substrate preference (5′-TC). Notably, the simultaneous replacement of loops 3 and 7 produced a hyperactive A3G mutant [108].

Deamination target selection varies among A3 proteins, with intrinsic preferences for specific motif sequences and substrate structures (Figure 2). For example, A3G protein targets C preceded by another C (5′-CC), whereas A1 and other A3 proteins target C preceded by T (5′-TC) [30,31,85,86,108,109,110,111]. However, targeting specific dinucleotide sites alone does not guarantee deamination. Some TC dinucleotide sites are not targeted by A3G protein, whereas others (known as hotspots) are preferentially deaminated [112,113]. This suggests that A3 proteins exhibit preferences beyond dinucleotide contexts that remain to be fully elucidated [114].

Both catalytically active domains [present in all single-domain A3 proteins and the C-terminal domains (CTDs) of double-domain A3 proteins] and catalytically inactive domains [found in N-terminal domains (NTDs) of double-domain A3 proteins] interact with nucleic acid substrates (Figure 2) (reviewed in [103,115,116]). The noncatalytic NTDs, despite lacking deaminase activity, regulate catalytic activity by guiding the proteins to active sites, promoting oligomerization, and enhancing ssDNA binding affinity (Figure 2) (reviewed in [116]). For instance, positively charged patches on the noncatalytic domains of A3B, A3F, and A3G proteins enhance deamination [117]. In contrast, RNA binding competitively regulates A3 proteins by rendering them catalytically inactive [118,119]. Cellular RNA and substrate ssDNA bind to the same A3G tryptic peptides; thereby, competition between ssDNA substrate and ssRNA to bind A3G limits its deamination capacity, as it was shown that RNA binding to A3G inhibits its further binding to ssDNA substrates as well as promotes A3G multimer dissociation from substrates [118,119]. Similarly, RNA binding alters the structure of A3H protein (via loops 1 and 7), reducing its deamination capacity [120]. It also attenuates the deamination rate of native A3B protein and diminishes its ability to induce double-stranded DNA breaks [121].

### 3.2. Cellular Factors Regulating the Enzymatic Activity of A3 Family Proteins

The enzymatic activity of A3 family proteins is regulated through interactions with various cellular RNAs and proteins (Figure 2). These interactions are mediated by their RNA-binding ability, leading to incorporation into large ribonucleoprotein complexes. Multiple reports have shown that A3 family proteins (at least A3C, A3F, A3G, and stable A3H, but not A3A) can form high molecular mass (HMM) complexes consisting of A3-binding RNAs, A3-binding proteins, and various RNA-binding proteins [117,122,123,124,125,126,127,128,129,130,131,132,133]. Notably, the enzymatic activity of cellular A3B, A3G, and A3H proteins is inactivated in HMM complexes but can be restored following RNase treatment [48,117,120,126]. For example, a study reported that virion-incorporated A3G is enzymatically inactivated by association with viral RNA, and degradation of viral RNA causes A3G-mediated hypermutation against viral cDNA intermediates [126]. Another study demonstrated that HIV-1 preferentially infects phytohemagglutinin (PHA)/interleukin-2 (IL-2)-activated CD4+ T cells due to promoting HMM A3G formation [127]. Additionally, a study showed that heat-shock protein (HSP) 70 stabilizes A3G protein [129]. HSP90 enhances the deamination activity of A3B, A3C, and A3G proteins during coexpression in human HepG2 liver cells and increases A3G’s C mutation efficiency in hepatitis B virus DNA [134]. Another cellular protein, ubiquitin-specific protease 49, stabilizes the A3G protein by removing the HIV-1 Vif ubiquitination mark, thereby enhancing its activity [135]. Furthermore, the depletion of exosome component 9, a component of the RNA exosome, results in reduced *A3G* mRNA expression levels in a cancer cell model [136].

The subcellular localization of A3 proteins is also influenced by cellular factors (Figure 2). Each A3 protein typically localizes to the cytoplasm, nucleus, or both (reviewed in [21,137]). A3A and A3C proteins exhibit cell-wide distributions [30,48,125,138,139,140], whereas A3B protein is primarily nuclear [30,48,120,138,139,140,141,142,143]. Notably, endogenous A3A protein localizes to the cytoplasm in primary CD14^+^ cells and interferon (IFN)-stimulated THP-1 cells, in contrast to overexpressed A3A protein in HEK293 and HeLa cells [30,125,139,144]. This difference in subcellular localization between endogenous and overexpressed A3A proteins suggests a regulatory mechanism governing its enzymatic activity. A3D, A3F, and A3G proteins are predominately cytoplasmic [30,120,125,130,139,140,142], whereas A3H protein shows variable localization patterns depending on its haplotype, with haplotype I (hapI) distributed throughout the cell and haplotype II (hapII) localized to the cytoplasm and nucleolus [120,140,145,146].

## 4. Restriction Activity of A3 Family Proteins Against HIV-1

Acquired immunodeficiency syndrome (AIDS) was first identified in 1981, and its causative agent, HIV-1, was confirmed in 1983. HIV-1 infection triggers the expression of host RFs and upregulates IFN-stimulated genes, including the A3 proteins (reviewed in [104,105,147]). A3 protein expression confers innate immune responses by inducing hypermutation of the viral genome, causing potentially lethal changes to the virus and further restricting infection. However, HIV-1 has developed a mechanism to evade this defense: the Vif protein recruits host cofactors to ubiquitinate A3 proteins for degradation, neutralizing this defense mechanism (reviewed in [2,18,23,34,42]). Although structural characteristics and cellular factors regulate the enzymatic activity of A3 proteins (refer to Section 3), this section discusses the interaction of A3 proteins with HIV-1, which determines HIV-1 infection outcomes.

### 4.1. Editing and Non-Editing Mechanisms for HIV-1 Restriction

HIV-1 is a well-documented target of A3 proteins (reviewed in [1,2,18,22]). Among these proteins, the A3G protein was the first found to inhibit viral replication in Vif-deficient HIV-1 (Figure 1) [26]. The HIV-1 restriction mechanisms employed by A3 proteins (mainly A3C I188, A3D, A3F, A3G, and A3H stable haplotypes) involve packaging into nascent virions from virus-producing cells, where they induce lethal C-to-U mutations in HIV-1 minus-strand cDNA intermediates in target cells (reviewed in [1,2,18,22]). Notably, A3 proteins are highly expressed in diverse immune cells and cell lines [29,34,132,141,148,149,150,151,152,153].

The antiviral activity of A3 proteins is also mediated through multiple deaminase-independent mechanisms (Figure 1) (reviewed in [2,17,22,154]). The roadblock model, a well-known deaminase-independent mechanism, involves A3G protein physically blocking viral reverse transcription and reducing the accumulation of reverse transcription products [34,35,36,37,39,40,66]. The direct interaction of A3G protein with HIV-1 reverse transcriptase also blocks reverse transcription [155,156]. Notably, the antiviral activity of A3F and A3H protein mainly arises in a deaminase-independent manner [120,157,158,159]. Importantly, A3G and A3F proteins interfere with viral genome integration by disrupting the structural integrity of the HIV-1 preintegration complex to inhibit proviral DNA integration into the host genome and by directly interacting with HIV-1 integrase to inhibit provirus formation [160,161] or compromising viral integration efficiency by affecting the processing of long extremities for viral long terminal repeats (LTRs) [162]. Additional non-editing activities of A3 proteins include the A3F protein, and, to a lesser extent, the A3G protein, remaining associated with the viral preintegration complex as it traffics into the host nucleus [163], altering proviral DNA integration site selection to avoid gene coding sequences and/or favoring integration into short interspersed nuclear elements, oncogenes, or transcription-silencing non-B DNA [160], potentially promoting more latent HIV-1 expression profiles (Figure 1).

Although A3G protein has been demonstrated to deaminate ssDNA, A3G-mediated deamination has not been observed in HIV-1 RNA or synthetic RNA oligonucleotides, ruling out RNA editing functions for A3G protein [85,87,164,165]. However, A3 proteins may mediate host modifications that facilitate cellular antiviral responses by editing host RNA. For instance, the A3A protein reportedly mediates widespread site-specific C-to-U RNA editing of cellular transcripts and host mRNA involved in proinflammatory (M1 phenotype) polarization of macrophages and in monocytes exposed to hypoxia and/or IFNs [166,167]. Additionally, transiently overexpressed A3G protein results in the editing of various host mRNAs in a HEK293T cell model [168,169]. A3G site specifically edits hundreds of genes [169], including those involved in HIV-1 replication, assembly, transcription, and infectivity, such as charged multivesicular body protein 4B [170], N-myristoyltransferase 1 [171], and RNA-binding motif protein 14 [172]. Further investigation is needed to determine the indirect effects of A3-mediated RNA editing on HIV-1 infection.

### 4.2. Counter-Defense Mechanisms Employed by Vif to Evade HIV-1 Restriction Activity by A3 Family Proteins

HIV-1 expresses the accessory protein Vif to counteract the antiviral activity of A3 proteins. Vif efficiently degrades A3 proteins in virus-producing cells and inhibits their packaging into nascent virions (reviewed in [2,18,23,34,42]). The primary function of HIV-1 Vif is to target A3 proteins for ubiquitination and proteasomal degradation by recruiting an E3 ubiquitin ligase complex composed of cullin 5 (CUL5), elongin B/C (ELOB/C), RING-box protein 2 (RBX2), transcription factor core-binding factor β (CBF-β), and Ariadne homolog 2 (ARIH2) (Figure 1) [34,173,174,175,176,177,178,179,180,181]. Structural studies have faced challenges in clarifying the costructures of Vif with full-length A3 proteins. However, a cryo-electron microscopy study revealed the structure of full-length human A3G protein bound to the HIV-1 Vif, CBF-β, ELOB, and ELOC (VCBC) complex, with RNA acting as a “molecular glue” for the A3G–Vif interaction, enabling Vif to repress the antiviral activity of A3G protein [176]. Other important Vif motifs are PPLP and its short downstream α-helix, α6. A recent study found that PPLP and α6 are critical to forming the functional VCBC complex in maintaining Vif-A3 interaction and are crucial for degrading A3 proteins [182].

HIV-1 Vif also mediates degradation-independent inhibition of A3 protein. For example, Vif inhibits A3G transcription by competing with Runt-related transcription factor (RUNX) and hijacking CBF-β, affecting *A3* gene expression, the regulatory domains of which are associated with RUNX [183,184]. Similarly, HIV-1 Vif induces translational inhibition of *A3G* mRNA via ribosome stalling at the 5′-untranslated region or shuttling *A3G* mRNA to ribonucleoprotein granules, thereby delaying or preventing translation [185,186]. Notably, HIV-1 Vif also induces G2/M cell cycle arrest, which potentiates HIV-1 replication in multiple cell lines [187,188,189,190,191,192,193]. Vif remodels the host phosphoproteome, efficiently depleting members of the PPP2R5 family of protein phosphatase 2A regulators, which are involved in G2/M progression regulation [191,193,194,195,196,197].

### 4.3. Natural Variations of A3 Family Proteins

In primary CD4^+^ T lymphocytes, up to five A3 proteins contribute to HIV-1 restriction (reviewed in [1,2,18,22]). HIV-1 Vif neutralizes this antiviral activity by targeting A3 proteins, a mechanism evolved to counteract the proteins’ effects (reviewed in [1,2,18,22]). However, A3 proteins exhibit genetic variations (Figure 2) that influence their antiviral activity against HIV-1 (reviewed in [198]). These variants may not necessarily correlate with Vif’s selective pressure but can impact the efficiency of A3 proteins in suppressing HIV replication. Moreover, a recent study showed that A3-induced mutations in the *env* and *gag-pol* region were correlating with *vif* diversity, suggesting that tolerance to such changes may benefit HIV-1 evolution [199].

A3C protein exhibits weak restriction activity against Vif-deficient HIV-1 [27,30,32,200], but the HIV-1 Vif targets A3C protein for proteasomal degradation [201,202], limiting its antiviral effects. A common A3C variant, characterized by a serine-to-isoleucine substitution at position 188, occurs frequently in African populations (around 10% prevalence) but globally at <2% [32,203]. This variant enhances A3C’s anti-HIV-1 activity in vitro [27,32]. Another rare variant, A3C S61P (<1% global frequency), also improves inhibition of Vif-deficient HIV-1 replication [200,204]. Structural analyses suggest that this variant exhibits improved interactions with ssDNA [200,204]. Despite these enhancements, A3C variants have limited mutagenic activity compared with other A3 family members [27,32,200,204], implying that they contribute to HIV-1 diversification through a lower mutation rate [204].

A3D protein displays superior antiviral activity compared with A3C protein, although its deaminase activity remains limited relative to that of A3F, A3G, and stable A3H haplotypes [30,203,205]. In a humanized mouse model, A3D protein is believed to play a role in HIV-1 diversification [206]. A3D variants, such as R97C and R238K, are more frequently observed in HIV-1-infected individuals compared to the general African population, with minor allele frequencies of 4.7% and 11.6%, respectively [203,207]. These variants exhibit markedly lower antiviral activity against Vif-deficient HIV-1 [203] but are highly sensitive to HIV-1 Vif degradation [203]. Although these common variants are less effective at restricting HIV-1 or HIV-2 compared with wild-type A3D protein [203,208], they share similar sensitivity to Vif.

A3F protein inhibits HIV-1 through deaminase-dependent and deaminase-independent mechanisms (Figure 1), with evidence suggesting that the latter is the predominant mode of inhibition [34,157,159,160]. A3F protein shows less mutagenicity compared to A3G protein but can drive HIV-1 evolution and confer drug resistance [206,209]. Common A3F variants include A108S, V231I, and Y307C [153,203,207,210]. The A3F Y307C variant is present at a low frequency in African and European populations (minor allele frequency < 5%) and absent in Asian populations [153,203,207]. This variant exhibits reduced antiviral activity and increased sensitivity to HIV-1 Vif [153,211]. The most frequent A3F polymorphisms are 108S/231I and 108A/231V [203,210]. These two single nucleotide polymorphisms (SNPs) show strong linkage disequilibrium in European and Asian populations but weaker linkage disequilibrium in mixed American and African populations [210]. Position 231 in the A3F protein contributes markedly to antiviral activity, with the A3F 231V variant being more stable and efficiently encapsidated into HIV-1 virions compared to the A3F 231I variant [210]. Interestingly, two A3F splice variants. A3FΔ2 and A3FΔ2–4, lack exon 2 and exons 2–4, respectively [212], and show lower expression levels and antiviral activity compared with wild-type A3F protein [212]. Notably, A3FΔ2 is resistant to Vif-mediated degradation, whereas A3FΔ2–4 is highly sensitive to Vif [212].

A3G, a potent antiviral protein, has been extensively investigated (reviewed in [1,2,18,22]). Among its many SNPs, the A3G H186R variant is particularly well-studied [203,213,214,215,216]. This variant is common in African American and African populations but rare in American European and European populations [203,213,215,216,217,218,219]. The H186R mutation is especially prevalent in the Zimbabwean population compared with the black South African (30%) and African American (37%) populations [219]. However, the antiviral activity of the A3G H186R variant is debated [203,214,215,216]. Another A3G variant, Q275E was found to be more common in a cohort of HIV-1-infected patients from Northern South Africa compared with African populations in the 1000 Genomes Project [207]. Duggal et al. showed that A3G H186R and Q275E variants exhibit antiviral activity similar to that of wild-type A3G protein, including in a dose-dependent manner [203].

A3H protein exists in two prominent haplotypes: stable and unstable [29,33,220,221]. These haplotypes are determined by four SNPs (positions 18, 105, 121, and 178) and one indel (position 15) in the *A3H* gene [29,33,220,221]. Classification of *A3H* haplotypes into stable and unstable is concluded by overexpression and pulse-chase experiments showing that 3 haplotypes yield proteins with relatively long half-lives (recognized as stable form), 1 haplotype produces a protein with weak stability, and further 3 haplotypes produce completely unstable proteins. A3H proteins produced from stable haplotypes are folded properly, resistant to degradation, and capable of HIV-1 restriction [220,221,222]. A3H haplotypes are further divided into four splice variants, namely SV154, SV182, SV183, and SV200, with SV200 being found only in stable haplotypes [33,223]. Interestingly, A3H hapI SV154 lacks deaminase activity, whereas other variants show strong activity, with slightly lower activity observed in A3H hap I SV200 [224]. According to the 1000 Genomes Project, 13 distinct A3H haplotypes exist [33]. The stable A3H hapII is predominant in African populations, whereas hapI is more common in other regions, including Europe and Asia [29,33]. Regardless of haplotype, all *A3H* mRNAs are detectable [29]. However, A3H unstable haplotypes are either difficult to detect (hapI) or undetectable, whereas stable haplotypes are easily detectable [28,29,33,220,221,225,226,227]. These A3H phenotypes align with their antiviral activity (Figure 2), where stable A3H haplotypes exhibit potent antiviral effects against HIV-1, whereas unstable haplotypes show reduced activity [28,29,33,220,221,225,226,227].

### 4.4. RNA Binding Capacity of A3 Family Proteins

During virion assembly, viral RNAs and host cell RNA polymerase III (pol III)–derived RNAs are packaged into virions (reviewed in [228,229]). RNA pol III–derived RNAs are noncoding RNAs essential for cellular functions (reviewed in [230,231]). The RNA pol III–derived RNAs commonly packaged into HIV-1 include 7SL RNA and Y RNA [123,232,233,234,235,236], with 7SL being a component of the signal recognition particle ribonucleoprotein complex and Y RNA being part of the Ro ribonucleoprotein complex (reviewed in [230,231]). These findings highlight the selective packaging of 7SL RNA and Y RNA by HIV-1 and suggest potential roles in viral assembly and replication.

The NTD of the A3G protein binds viral and cellular RNAs [35,36,37,38], whereas the A3F protein uses both its NTD and CTD for packaging [237]. These domains enable the incorporation of A3F and A3G proteins into virions, where they exert their antiviral activity (reviewed in [238]). Several studies have shown that 7SL RNA is highly enriched in HIV-1 virions, with A3F and A3G proteins preferentially binding 7SL RNA over Y RNA [232,233,239]. Notably, 7SL RNA is pivotal for the efficient incorporation of A3F and A3G proteins into virions [232,233,234,235]. Furthermore, the mutants A3F W126A and A3G W127A showed reduced 7SL RNA binding, exhibited poor RNA packaging, and thereby impaired antiviral activity [232,233], highlighting the relevance of 7SL RNA-mediated A3 packaging to their antiviral function. However, it has been indicated that 7SL RNA is not essential for the packaging of A3F and A3G proteins into virions [234]. Hence, further extensive studies will need to conclude whether 7SL RNA-mediated A3 packaging is important for their antiviral function.

A3F and A3G proteins share similar packaging mechanisms through interaction with the nucleocapsid (NC) domain [38,235,240,241,242,243,244,245,246,247]. Studies have shown that A3G’s interaction with the NC domain is RNA-dependent [240,243,246,248], whereas others have proposed that the interaction is direct [241,247]. Both A3F and A3G proteins associate with viral RNA sequences enriched in G and/or adenine (A), which are not scanned by ribosomes during translation [235]. They recognize unpaired 5′-AA motifs and, to a lesser extent, 5′-GA motifs [242]. A3 proteins also mimic the RNA-binding specificity of the NC domain [235], with binding to this domain ensuring A3G’s concentration in the viral core of mature HIV-1, near the reverse transcription complex. A3F and A3G proteins bind 3′-AA/GA motifs through an aromatic/hydrophobic pocket in the noncatalytic domain and 5′-AA/GA motifs via an aromatic/hydrophobic groove between the noncatalytic and catalytic domains [242,249].

RNA binding markedly increases the likelihood of A3 proteins being packaged into virions, indirectly supporting their antiviral activity. Cryo-electron microscopy studies have revealed that RNA acts as a “molecular glue” in the Vif–A3G interaction [176,180]. This is due to the negative electrostatic potentials on the Vif binding patches of A3G protein, whereas the RNA binding site is positively charged [181]. When RNA binds, the A3G–RNA complex displays an expanded region with negative electrostatic potentials [181], facilitating interactions with the positively charged Vif surfaces [181]. Thus, RNA binding enhances electrostatic complementarity between A3G and Vif, promoting their assembly [181]. This highlights the role of RNA binding in benefiting Vif.

### 4.5. Post-Translational Modulation of A3 Family Proteins

Phosphorylation regulates the antiviral activity of A3 proteins (Figure 2) [128]. For instance, phosphorylation at the A3G Thr32 residue reduces its degradation by diminishing its affinity for HIV-1 Vif [128]. This modulation allows A3 proteins to bind distinct nucleic acid substrates and specific motifs, influencing their catalytic activity [112,113]. Cellular mechanisms also regulate A3 protein expression (Figure 2). Proinflammatory cytokines, such as IFN-α/β, tumor necrosis factor (TNF)-α, IL-6, and IL-1β, are known to enhance A3 expression (reviewed in [147,250]). IFNs, for example, increase *A3A* and *A3G* mRNA levels along with their protein expression in myeloid cells, such as monocyte-derived macrophages and dendritic cells [144,148,251,252,253,254]. In HIV-1-infected monocyte-derived macrophages, IFN-α treatment boosts A3A protein expression and activity, marked by increased G-to-A editing and reduced viral DNA accumulation [254]. Additionally, cytokines that regulate macrophage polarization toward the M1 (IFN-γ and TNF-α) or M2 (IL-4) phenotypes modulate A3A and A3G protein expression [255]. Alternative splicing also influences A3 function through isoform generation; this is exemplified in the A3H protein, where isoforms exhibit nonfunctional, maintained, and enhanced antiviral activity [33,223].

### 4.6. Impact of A3 Family Proteins on AIDS Progression

Naturally occurring A3 variants not only influence antiviral activity but also correlate with AIDS progression [28,213,216,256,257,258]. The A3F 231V allele, for example, is associated with lower viral loads and slower AIDS progression [257]. Additionally, the A3G H186R variant has been linked to a decline in CD4^+^ T-cell counts [213,216] and accelerated AIDS progression [216,256]. However, other studies suggest that the A3G H186R variant does not markedly affect these parameters [217,259], possibly due to population genetic diversity. Furthermore, HIV-1-infected individuals with stable A3H haplotypes show slower AIDS progression [28,258]. Although the genetic variations of A3 family proteins are among the factors that affect AIDS progression, the genetic diversity of other host and virus factors associated with the antiviral activity of A3 family proteins should also be considered.

Several factors impact the mutagenic activity of A3 proteins against HIV-1 (reviewed in [22,260,261,262]). Lethal HIV mutagenesis is counter-selected, whereas moderate A3-induced mutations generate sublethal changes that enhance viral diversity and immune evasion [206,263,264,265,266,267,268,269,270,271,272]. This has been confirmed by constructing phylogenies of A3-induced hypermutated proviruses of HIV-1-infected individuals on antiretroviral therapy (ART), showing hypermutated viruses can persist for decades and may follow different evolutionary dynamics compared to intact proviruses [273]. Mutations in A3-preferred motifs often occur in HIV-1 epitopes targeted by cytotoxic T lymphocytes, promoting immune escape and reducing CD8^+^ T-cell responses [270,272]. Boichard et al. suggested that overexpression of the programmed cell-death ligand is correlated with A3 proteins and may contribute to immune exhaustion, potentially leading to AIDS [274]. Recently, it has been demonstrated that HIV-1 selectively packages intact genomic RNA despite A3G-induced hypermutation in cDNA, revealing a decoupling of G-to-A hypermutation from viral infectivity [275]. This may highlight a mechanism of HIV-1 for maintaining functional genomes under antiviral pressure and conserved replication.

Of note, A3-mediated HIV-1 proviral hypermutation not only produces defective proviruses but also involves positions scored for drug resistance and are referred to as APOBEC-context drug resistance mutations (AC-DRMs) [276]. Defective *pol* sequences are shown to harbor most of the AC DRMs. However, they are not associated with HIV-1 DNA levels in infected individuals, and their impact on clinical settings is not confirmed [277].

## 5. A3-Related Determinants of HTLV-1 Infection

Infection with the Deltaretrovirus HTLV-1 generally results in lifelong asymptomatic carriers in the majority of infected individuals, whereas approximately 5% of cases progress to develop adult T-cell leukemia/lymphoma (ATL) or HTLV-1-associated myopathy (HAM) (reviewed in [44,278,279]). However, the mechanisms driving progression toward either of these pathologies remain poorly understood. HTLV-1 evolved from the simian retrovirus simian T-cell leukemia virus type 1 through cross-species transmission to humans approximately 20,000–50,000 years ago (reviewed in [278,280,281,282]). Despite primarily targeting CD4^+^ T lymphocytes, HTLV-1 can also infect other immune cells, including CD8^+^ T cells and myeloid cells, especially dendritic cells, which can become productively infected and transmit the virus to CD4^+^ T cells (reviewed in [283]). Once integrated into the host genome, HTLV-1 maintains chronic infection through clonal expansion of infected CD4^+^ T cells, limiting genetic variability (as reverse transcription is not involved) and reducing exposure to antiviral factors [284]. However, low-level viral spread via cell-to-cell transmission, including through viral synapses, protrusions, and tunneling nanotubes, has also been reported (Figure 3) [285]. Unlike HIV-1 infection of CD4^+^ T cells, which results in the massive release of new viral particles and cell death, HTLV-1 infection induces rapid cessation of viral particle production and promotes CD4^+^ T-cell proliferation, transformation, and immortalization (reviewed in [286,287]). Therefore, both clonal expansion and cell-to-cell transmission suggest that host factors play an intrinsic role in restricting HTLV-1 infection (reviewed in [288]).

The 9-kb HTLV-1 genome encodes for *Gag*, *Pol*, *Env*, *p12*, *p13*, *Rex*, *Tax,* and *HTLV-1 bZIB factor* (*HBZ*) genes. These are transcribed from the 5′-LTR, except for HBZ, which is encoded on the proviral minus strand and transcribed from the 3′-LTR (reviewed in [44,45,283]). Two oncogenic genes, *Tax* and *HBZ*, produce viral regulatory proteins that mediate the pathogenicity of HTLV-1 infection (reviewed in [43,44,45]). Tax protein promotes viral transcription and is closely associated with immune dysregulation in patients with HAM, inducing diverse cellular gene expressions through activation of the nuclear factor kappa B (NF-κB) and cAMP response element binding protein/activating transcription factor (CREB/ATF) pathways, driving neoplastic transformation (reviewed in [45]). As Tax is a major target for cytotoxic T lymphocytes, its expression is tightly controlled to ensure the survival of HTLV-1-infected cells and evade the host immune response [289]. *HBZ* mRNA is ubiquitously expressed in HTLV-1-infected cells, including in peripheral blood mononuclear cells from infected individuals and ATL cells, where it promotes the growth and survival of leukemic cells [290]. HBZ has also been shown to interact with the CREB/ATF pathway, selectively inhibit the NF-κB pathway, and suppress Tax-mediated viral transcription (reviewed in [45,291]).

### 5.1. A3–HTLV-1 Interplay: Restriction Versus Resistance

HTLV-1 preferentially targets CD4^+^ T cells, which express several A3 family proteins [29,141,148,149,151,153]. However, the antiviral activity of A3 proteins against HTLV-1 remains controversial. Overexpression studies have shown that multiple A3 proteins (A3A, A3B, and A3H hapII) can restrict HTLV-1 infectivity through deaminase-dependent and -independent mechanisms [292,293,294]. Additionally, it has been proposed that nonsense mutations in the HTLV-1 genome, induced by the A3G protein in asymptomatic carriers and patients with ATL, could allow the virus to evade the host immune response [295]. A3-mediated editing of the HTLV-1 genome during viral reverse transcription has been confirmed (Figure 3), where A3-targeted motifs are underrepresented (depleted) in the *HBZ* gene, with similar findings in *Gag*, *Pol*, and *Tax*, indicating corresponding A3 editing activity [50]. HTLV-1 is particularly susceptible to A3G-mediated deamination during reverse transcription, as demonstrated by a study analyzing HTLV-1 provirus sequences from asymptomatic carriers and patients with ATL, which revealed A3G-preferred G-to-A mutations in around 50% of cases [295]. A comparative study of HTLV-1-infected asymptomatic carriers and HTLV-2-infected individuals showed that the A3G protein frequently generates G-to-A mutations in the HTLV-1 provirus, whereas these mutations are rare in the HTLV-2 provirus [296]. Deep sequencing of full HTLV-1 proviruses from asymptomatic carriers indicated that G-to-A mutations represented 73% of all detected mutations, with 87.1% of these being GG-to-AG mutations, which are a preferred target for A3G protein [296].

Notably, HTLV-1 appears to be relatively resistant or poorly susceptible to A3 proteins (Figure 3), as demonstrated via experiments showing that HTLV-1 is not efficiently restricted by A3D, A3F, or A3G proteins [38,292,294,297]. Unlike HIV-1, HTLV-1 does not encode a viral product, such as Vif, to mediate A3 antagonism, and it is apparently unable to degrade A3 proteins in cell cultures [298]. Consistent with this finding, hyper-edited HTLV-1 sequences are rare, with estimated frequencies of 0.1–5.0% in vitro [38,292]. However, despite increased A3G expression in patients with HTLV-1, this was not correlated with clinical status or proviral load [299]. The resistance of HTLV-1 to A3-mediated restriction is believed to be due to lower levels of A3G encapsidation [297]. A direct resistance mechanism to A3G protein has been described in HTLV-1, functioning via a *cis*-acting exclusion mechanism, which involves an acidic region in the C-terminus of the HTLV-1 NC domain. This mechanism leads to reduced A3G packaging efficiency in HTLV-1 particles compared with HIV-1 Vif-deficient virus-like particles and is not attributed to a viral accessory protein [297].

### 5.2. Factors Determining A3–HTLV-1 Interaction Outcomes

The viral replication strategy plays a major role in determining the effectiveness of A3-mediated restriction on HTLV-1 infection. HTLV-1 exhibits low-level replication and relies on the clonal expansion of infected cells, resulting in a reduced rate of *de novo* infection as well as cell-to-cell viral spread (reviewed in [43,44,45]). This infrequent replication, combined with reverse transcription in HTLV-1, substantially decreases the opportunities for A3 proteins to edit the viral genome [297]. Additionally, the genetic diversity of HTLV-1 is lower than that of HIV-1, particularly in the context of their respective *env* genes, which further suggests that HTLV-1 is more resistant to the antiviral activity of A3 proteins [300,301].

The HTLV-1 genome is notably GC-rich compared with the HIV-1 genome [302]. Despite this, the relatively low occurrence of G-to-A mutations in the HTLV-1 genome indicates that HTLV-1 is less susceptible to the mutagenic activity of A3 proteins. Although the HTLV-1 genome does not encode Vif or a Vif-like protein, it can still resist the antiviral activity of A3 proteins (particularly A3G), at least in vitro [297]. Moreover, although endogenous and overexpressed A3G proteins can be packaged into HTLV-1 virions produced in HEK293 and MT-2 cells, the frequency of G-to-A mutations remains low [292]. Derse et al. demonstrated that HTLV-1 diminishes A3 protein packaging (Figure 3) through a peptide motif in the C-terminal domain of the NC [297]. Nevertheless, A3-mediated mutations in HTLV-1 may be influenced by the genetic variability of A3 proteins. For example, higher frequencies of G-to-A mutations were observed in a small group of HTLV-1-associated disease cases harboring two rare A3G variants relative to the remaining study population lacking these rare variants [303].

## 6. Conclusions

The contribution of A3 family proteins to retrovirus restriction underscores their essential role as key host RFs in humans. These proteins mediate antiviral activity by inducing lethal mutations, leading to virus restriction. However, the overall restriction activity of A3 family proteins is governed by multiple factors, including structural elements, substrate selection, and cellular regulators, all of which markedly influence their editing and non-editing functions. HIV-1 counteracts A3-mediated restriction primarily through Vif, which triggers proteasomal degradation of A3 proteins. In contrast, HTLV-1 employs a unique dissemination strategy that minimizes exposure to A3 proteins by avoiding the production and release of new viral particles. Nevertheless, A3-mediated restriction is not completely circumvented, as evidenced by the presence of A3 protein footprints on HIV-1 and HTLV-1 genomes in infected individuals. Therefore, understanding the determinants of retroviral infection mediated by A3 proteins is essential for determining the outcomes of infection and for exploring A3 proteins as potential therapeutic tools via the manipulation of their mutagenic activity.
